# Understanding the potential of digital therapies in implementing the standard of care for depression in Europe

**DOI:** 10.1192/j.eurpsy.2023.2453

**Published:** 2023-10-24

**Authors:** Philippe Courtet, Odile Amiot, Enrique Baca-Garcia, Lara Bellardita, Giancarlo Cerveri, Anne-Hélène Clair, Diego De Leo, Dominique Drapier, Eric Fakra, Francis Gheysen, Lucas Giner, Ana Gonzalez-Pinto, Gualberto Gussoni, Emmanuel Haffen, Laurent Lecardeur, Fermin Mayoral-Cleries, Francesco Saverio Mennini, Pilar A Sáiz, Eduard Vieta, Diego Alberto Hidalgo, Umberto Volpe

**Affiliations:** 1 Department of Emergency Psychiatry and Acute Care, Lapeyronie Hospital CHU Montpellier, Institut de Génomique Fonctionnelle, Université de Montpellier, CNRS, INSERM, Montpellier, France; 2 GH Paul Guiraud, Boulogne Billancourt, France; 3 Department of Psychiatry, University Hospital Jimenez Diaz Foundation, Madrid, Spain; 4 Department of Psychiatry, University Hospital Rey Juan Carlos, Mostoles, Spain; 5 Department of Psychiatry, General Hospital of Villalba, Madrid, Spain; 6 Department of Psychiatry, University Hospital Infanta Elena, Valdemoro, Spain; 7 Department of Psychiatry, Madrid Autonomous University, Madrid, Spain; 8 Centro de Investigacion en Salud Mental (CIBERSAM), Carlos III Institute of Health, Madrid, Spain; 9 Department of Psychiatry, Centre Hospitalier Universitaire de Nîmes, Nîmes, France; 10 Maggiolina and Città Studi Center, Milan, Italy; 11 Department of Mental Health, Addiction Hospital, Lodi, Italy; 12 Institut du Cerveau – Paris Brain Institute – ICM, Sorbonne Université, INSERM, CNRS, Paris, France; 13 Department of Psychology, Primorska University, Koper, Slovenia; 14 Centre Hospitalier Guillaume régnier, Pôle Hospitalo universitaire de psychiatrie, CIC Rennes INSERM, Rennes, France; 15 Saint Étienne University Hospital, University Jean Monnet, Saint Étienne, France; 16 PsyR2 Team, Centre de Recherche en Neurosciences de Lyon (CRNL), INSERM U1028, CNRS UMR 5292, Université Jean Monnet Saint Etienne, Université Lyon 1, Saint-Étienne, France; 17 Compassionate Mind Foundation France, Caen, France; 18 Department of Psychiatry, Universidad de Sevilla, Seville, Spain; 19 BIOARABA, Department of Psychiatry, Hospital Universitario de Alava, UPV/EHU, CIBERSAM, Vitoria, Spain; 20 Clinical Research Department, Italian Scientific Society of Internal Medicine (FADOI), Milan, Italy; 21 UR LINC, Service de psychiatrie de l’adulte, CIC-1431 INSERM, CHU de Besançon, Université de Franche-Comté, Besançon, France; 22 DueL, Nice, France; 23 Department of Mental Health, Regional University Hospital of Málaga, Biomedical Research Institute of Malaga (IBIMA), Málaga, Spain; 24 EEHTA-CEIS, DEF Department, Faculty of Economic, University of Rome Tor Vergata, Roma, Italy; 25 Institute for Leadership and Management in Health, Kingston University, London, UK; 26 Department of Psychiatry, Instituto de Investigación Sanitaria del Principado de Asturias (ISPA), University of Oviedo, Oviedo, Spain; 27 Instituto de Neurociencias del Principado de Asturias (INEUROPA), Mental Health Services of the Principality of Asturias (SESPA), Oviedo, Spain; 28 Hospital Clinic, Institute of Neurosciences, University of Barcelona, IDIBAPS, CIBERSAM, Barcelona, Spain; 29 Bipolar Disorders Unit, Hospital Clinic, Institute of Neurosciences, University of Barcelona, IDIBAPS, CIBERSAM, Barcelona, Spain; 30 Clinical Psychiatry Unit, Department of Clinical Neurosciences, Università Politecnica delle Marche, Ancona, Italy

**Keywords:** Digital therapeutics, DTx, depression, Deprexis

## Abstract

Depressive disorders represent the largest proportion of mental illnesses, and by 2030, they are expected to be the first cause of disability-adjusted life years [1]. The COVID-19 pandemic exacerbated prevalence and burden of depression and increased the occurrence of depressive symptoms in general population [2]. The urgency of implementing mental health services to address new barriers to care persuaded clinicians to use telemedicine to follow patients and stay in touch with them, and to explore digital therapeutics (DTx) as potential tools for clinical intervention [2]. The combination of antidepressants and psychotherapy is widely recommended for depression by international guidelines [3] but is less frequently applied in real-world practice. Commonly used treatments are pharmacological, but while being effective, some aspects such as adherence to the drug regimen, residual symptoms, resistance, lack of information, and stigma may hinder successful treatment. In case of less severe depression, standalone psychological therapies should be the first-line treatment option [3], but access to trained psychotherapists remains inequitable. DTx are evidence-based therapies driven by software programs to treat or complement treatment of a specific disease. DTx are classified as Medical Devices, and given their therapeutic purpose, they need to be validated through randomized controlled clinical trials, as for drug-based therapies. In the last 10 years, studies of digital interventions have proliferated; these studies demonstrate that digital interventions increase remission rates and lower the severity of depressive symptoms compared with waitlist, treatment as usual, and attention control conditions [4]. Despite the efficacy demonstrated in clinical trials, many of these tools never reach real-life patients; thus, it might be necessary to implement DTx in the public health system to expand access to valid treatment options. In this framework, DTx represent a good opportunity to help people with depression receive optimal psychotherapeutic care [5].

Depressive disorders represent the largest proportion of mental illnesses, and by 2030, they are expected to be the first cause of disability-adjusted life years [1]. The COVID-19 pandemic exacerbated prevalence and burden of depression and increased the occurrence of depressive symptoms in general population [2]. The urgency of implementing mental health services to address new barriers to care persuaded clinicians to use telemedicine to follow patients and stay in touch with them, and to explore digital therapeutics (DTx) as potential tools for clinical intervention [2]. The combination of antidepressants and psychotherapy is widely recommended for depression by international guidelines [3] but is less frequently applied in real-world practice. Commonly used treatments are pharmacological, but while being effective, some aspects such as adherence to the drug regimen, residual symptoms, resistance, lack of information, and stigma may hinder successful treatment. In case of less severe depression, standalone psychological therapies should be the first-line treatment option [3], but access to trained psychotherapists remains inequitable. DTx are evidence-based therapies driven by software programs to treat or complement treatment of a specific disease. DTx are classified as Medical Devices, and given their therapeutic purpose, they need to be validated through randomized controlled clinical trials, as for drug-based therapies. In the last 10 years, studies of digital interventions have proliferated; these studies demonstrate that digital interventions increase remission rates and lower the severity of depressive symptoms compared with waitlist, treatment as usual, and attention control conditions [4]. Despite the efficacy demonstrated in clinical trials, many of these tools never reach real-life patients; thus, it might be necessary to implement DTx in the public health system to expand access to valid treatment options. In this framework, DTx represent a good opportunity to help people with depression receive optimal psychotherapeutic care [5].

The pandemic offered the opportunity to accelerate the development of digitally delivered mental health services to manage patients with depressive disorders. It has been demonstrated that during the pandemic, mental health DTx have improved subjective well-being and the rate of clinical improvement of depressive symptoms [6]. At the European level, no specific legal regulation exists for the use of DTx in mental health, and on the national level, the situation is very much heterogeneous. While some countries start to explore solutions (e.g., Spain and Italy), others already have pathways for DTx assessment and reimbursement (e.g., Germany, the UK, and France).

Taking the opportunity of the entry of digital solutions in the European market, experts aimed at building knowledge on DTx for depression and providing useful advice for their use in clinical practice. Pros and cons of the potential adoption of DTx to treat depression have been evaluated in three European countries currently in a phase of digital development (i.e., France, Italy, and Spain). For each country involved in this study, a panel of psychologists and psychiatrists in the field of depressive disorders had access to a depression-focused Internet intervention called Deprexis®, which is specifically named in the National Institute for Health and Care Excellence (NICE) guidelines [7] and has strong evidence of effectiveness in reducing depressive symptoms over a wide range of initial symptom severity [8]. The participants filled out a SWOT template to analyze the strengths (S), weaknesses (W), opportunities (O), and threats (T) of the DTx experience they had. Data were gathered, analyzed, and initially discussed by the participants in each country. Results were then critically reviewed and discussed collectively by three experts with shared expertise in the clinical management and “digital” treatment of depression. The panel worked toward a mutual agreement, and the discussion led to a consensus on what aspects must be considered DTx pros and cons and what scenarios the adoption of DTx would open to the care of depression in Europe. The project took place from December 2021 to December 2022.

Experts agreed that there are several pros related to DTx for depression (Table 1). The possibility of following a psychotherapy program accessible online, available at any time, and with no geographical restriction allows patients to receive psychological support at the exact moment they need it and reduces the fear of stigma. The simplicity of access and use of the tool can facilitate the acceptability of patients in their treatment actively involving them in a home-based psychological support. Moreover, this type of therapy can be used alone in individuals with first episodes of non-severe depression, or in combination with face-to-face psychotherapy and/or antidepressants to guarantee broader mental care assistance to patients with depression. DTx, by increasing self-management, promote patients’ empowerment and thus may improve long-term outcomes. The cons that emerged from the panel discussion (Table 1) are mainly related to difficulties in engaging patients. Cognitive alterations, emotional dysregulation, cognitive negativity bias, fatigue, and lack of motivation along with the little interaction with the tool can limit the active involvement of the patient and cause low adherence after download. Moreover, the low possibility to monitor the clinical state of the patient in real time prevents a reactive response in case of risk situations. For all these reasons, experts agreed that this type of mental healthcare delivery might be more useful and appropriate for treating mild and moderate but not severe depression.

**Table 1.** Combined results: common aspects and discrepancies

Common aspects		Discrepancies
Pros	Cons	
Broad accessibility	Low adherence after download (better for mild depression)	Level of digitalization
Simple and effective communication	Little interaction	Perception of DTx as complementary/supplementary to psychotherapy
Provides support in moments when therapists are not available	Limited possibilities for tracking and monitoring clinical state	Cultural habits
Active involvement in home-based psychological support	No contact with professionals in emergency situations	Reimbursement pathways
Regulatory-friendly (Class I medical device)		
Complementary to psychotherapy and pharmacological therapy		
Cost-effectiveness		

Advantages and limitations of DTx for depression identified here are those usually attributed to such tools in the scientific literature. However, the comparison of the analyses done in Spain, France, and Italy revealed some differences and specificities among countries worth considering (Table 1). According to the Digital Economy and Society Index 2022, there is a wide difference among European countries in terms of overall digital performance [9]. Differences in the state of IT infrastructure and the level of digital skills of people affect the degree of accessibility to DTx. A good level of digitalization in a country drives confidence in the use of DTx to treat depression and consequently also influences the clinicians’ perception of DTx as complementary rather than supplementary to psychotherapy. The discrepancies related to different regional contexts that emerged from this work (Table 1) are relevant aspects to think of when implementing digital solutions for mental care in Europe. Even if DTx are designed with a customization approach for the patient’s experience, they still often lack to consider country-specific cultural differences (i.e., customs and traditions).

DTx represent an opportunity to improve standard of care for depression in clinical practice and real-world setting. Digitally delivered interventions help patients’ clinical improvement across a broad range of symptom types and severity and can be combined with other forms of treatment for depression [8]. For patients with initial mild to moderate symptoms of depression, this type of intervention may represent the first access to psychological therapies [3]. Thus, promoting early intervention on patient symptoms, also via DTx, would reduce the burden of patients who develop severe depression.

The increasing prevalence of depressive disorders and the reduction in Cognitive Behavioral Therapy practitioners in many European countries necessitate a rethinking of mental healthcare, exploring new prevention and treatment approaches. Thus, also, general practitioners might be involved in prescribing DTx as part of therapeutic arsenal for patients with less severe depression.

Despite the need to meet the high demand for mental care and the tremendous potential of DTx for depression, there are still some barriers to the broad dissemination of these interventions in Europe. Local adaptation is possible; however, there is need for European guidelines regarding the use of DTx to treat depression. Defined assessment criteria for DTx certification and robust indications on the use of DTx in the standard of care for depression would support reimbursement of these interventions in more countries. Most DTx involve charges to users and reimbursement pathways are important to ensure equitable access to therapy. At the global level, Europe is leading the way in DTx reimbursement models; however, the situation is very heterogeneous. Legal regulation on DTx by the European Medicines Agency and the European Commission is needed to integrate digital healthcare into the public healthcare system in more countries.

COVID-19 pandemic favored speeding digitalization process in Europe, digital transformation of healthcare, and encouraged the use of digital therapies highlighting the value of DTx in depression care pathways. The Council of the European Union recognizes that digitalization development is changing the way health services are delivered and calls for a comprehensive EU mental health strategy. The NICE guidelines recommend DTx for the treatment of depression; Deprexis® is one of the three DTx suggested [7]. The European Commission has set objectives in the European skills agenda and the digital education action to guarantee that 70% of adults (aged 16–74) have at least basic digital skills by 2025 [10]. These actions will contribute to increasing the average age of people using digital technologies and therefore also covering the ages where symptoms of depression are more frequent.

The alarming burden of depressive disorders and increasing need to adopt new ways and tools to provide mental health therapies call for the resolution of digital and legal discrepancies across European countries to rapidly implement DTx in the standard of care for depression.
